# Differential correlation between time in range and eGFR or albuminuria in type 2 diabetes

**DOI:** 10.1186/s13098-023-01071-4

**Published:** 2023-05-05

**Authors:** Xuguang Jin, Xinyi Yang, Yixin Xu, Jingjing Liang, Chunyan Liu, Qingyu Guo, Wei Wang, Zhouqin Feng, Yanyu Yuan, Hui Zhou, Zhen Zhang, Wenwen Jiang, Yue Liang, Bin Lu, Jiaqing Shao, Yong Zhong, Ping Gu

**Affiliations:** 1 Medical School, Affiliated Jinling Hospital, Department of Endocrinology, Nanjing University, 305 East Zhongshan Road, Nanjing, 210002 Jiangsu China; 2 Department of Endocrinology, Jinling Hospital, First School of Clinical Medicine, Southern Medical University, Nanjing, China; 3 Department of Endocrinology, the affiliated Jinling Hospital of Nanjing Medical University, Nanjing, Jiangsu China

**Keywords:** Albuminuria, Hypoglycemia, Glomerular filtration rate, Time in range, Nocturnal time in range, Continuous glucose monitoring, Hemoglobin A1C, Type 2 diabetes mellitus

## Abstract

**Introduction:**

As a CGM-derived indicator, ‘time in range’ (TIR) is emerging as a key indicator for accurate assessment of glycaemic control. However, there is few report focusing on the correlation of TIR with albumuria and renal fuction. The aim of this work was to investigate whether TIR, as well as nocturnal TIR and hypoglycaemic events is related to the presence and severity of albuminuria and decrease of eGFR in type 2 diabetes.

**Research design and methods:**

A total of 823 patients were enrolled in this study. All patients received continuous glucose monitoring, TIR indicating the percentage of time that blood glucose was in the range of 3.9–10.0 mmol/L. The Spearman analysis was applied to analyze the relationship between TIR (or nocturnal TIR) and ACR. Logistic regression was used to explore whether TIR (or nocturnal TIR) is an independent risk factor for albuminuria.

**Results:**

The prevalence of albuminuria decreased with increasing TIR quartiles. Binary logistic regression revealed that TIR as well as nocturnal TIR was obviously related to the presence of albuminuria. Multiple regression analysis found that only nocturnal TIR was obviously related to the severity of albuminuria. In our study, eGFR was significantly associated with the number of hypoglycemic events.

**Conclusions:**

In T2DM patients, TIR and nocturnal TIR is associated with the presence of albuminuria independent of HbA1c and GV metrics. Nocturnal TIR shows better correlation than TIR. The role of TIR especially nocturnal TIR in the evaluation of diabetes kidney disease should be emphasized.

## Introduction

According to the Diabetes Control and Complications Trial (DCCT) and the UK Prospective Diabetes Study (UKPDS) [1, 2], hemoglobin A1C is the gold standard for assessing glycaemic control for decades and is strongly associated with the risk of long-term diabetic complications. However, as an indirect measure of average blood glucose levels over three months, HbA1c is considered to have some limitations [3]. HbA1c does not provide details about hypoglycaemia or hyperglycaemia and does not reflect glycemic variability [4]. HbA1c also has limitations in interpreting the risk of chronic complications of diabetes. For instance, HbA1c accounted for only 11% of the variation in risk of diabetic retinopathy observed in the Diabetes Control and Complications Trial (DCCT) [5].

Continuous glucose monitoring (CGM) provides an accurate reflection of an individual’s blood glucose status throughout the day. Compared to glycosylated haemoglobin, CGM technology can better reflect blood glucose variability. As a CGM-derived indicator, ‘time in range’ (TIR) is simple, intuitive and responsive to treatment and lifestyle changes, and has become a key indicator for assessing glycaemic control. It is negatively correlated with glycated haemoglobin and the American Diabetes Association 2021 guidelines [6] stated that it can be used to assess glycaemic control and might be an acceptable endpoint for future clinical trials. Several different studies [7] have reported that time range (TIR) is associated with the risk of microvascular complications and can predict the risk of future diabetic complications. Furthermore, according to the study [8], average blood glucose levels at night, but not daytime blood glucose values or glucose variability, were independently associated with the degree of vascular remodelling. This suggests that nighttime blood glucose may be a better indicator of the risk of diabetic complications than full-day blood glucose status. Circadian rhythms have received increasing attention in recent years with the award of the 2017 Nobel Prize to Young MW et al. [9] for their discoveries of molecular mechanisms controlling the circadian rhythm. Circadian rhythms also exist for various hypoglycemic hormones and baseline blood glucose levels, and studies have shown that baseline pre-meal glucose levels in animals and healthy humans show circadian rhythms under a regular light/dark cycle, with a trough during sleep and a peak during wakefulness [10–12]. Nevertheless, the clinical significance of circadian rhythms in blood glucose is not clear.

Diabetic kidney disease (DKD) is a common chronic microvascular complication of diabetes that is now a major cause of CKD and end-stage renal disease, manifested mainly by a urinary albumin/creatinine ratio (UACR) ≥ 30 mg/g and/or an estimated glomerular filtration rate (eGFR) < 60 ml-min -¹-(1.73 m²)-¹ that persists for more than 3 months. In early screening, a random urine measurement of UACR is recommended to reflect urinary albumin excretion. TIR was found to be strongly associated with albuminuria in type 2 diabetes [13]. Furthermore, respective study found that each 10% treatment-induced increase in TIR was associated with 18% reduction in albuminuria in patients with T1D [14]. However, there is little report on the impact of nocturnal TIR and albuminuria in T2D.

The aim of this work was to investigate whether TIR measured by CGM, especially nocturnal TIR and hypoglycaemic events is related to the presence and severity of albuminuria and decrease of eGFR in type 2 patients with diabetes.

## Research design and methods

### Participants

A total of 823 patients (aged ≥ 18 years) with T2DM admitted to the Department of Endocrinology, Jinling Hospital, Nanjing University from April 2018 to July 2020 were recruited, all of whom met the 1999 WHO diagnostic criteria for type 2 diabetes mellitus. Exclusion criteria included (1) patients with acute complications of diabetes, acute stress such as trauma, surgery and severe infections, severe respiratory disease, malignant disease, pregnancy; (2) patients with definite hepatic disease; (3) patients with narcotic and psychotropic drugs, and a recent history of alcoholism; (4) using the Chronic Kidney Disease Epidemiology Collaboration (CKD-EPI) formula to calculate, patients with an estimated glomerular filtration rate (eGFR) of less than 30 ml/min/1.73m2. The study was supported by the local ethics committee.

### Clinical and biochemical measurements

General clinical information and physical examination such as age, sex, duration of diabetes, hypertension, diabetic retinopathy and diabetic kidney disease were recorded. Height, weight, systolic blood pressure (SBP) were measured. Body mass index (BMI) was computed. Biochemical measurements such as blood and urine samples were tested after a 12-hour overnight fast. Hemoglobin A1C (HbA1c), total cholesterol (TC), triglyceride (TG), high-density lipoprotein (HDL), low-density lipoprotein (LDL), blood urea nitrogen (BUN), and serum creatinine (SCr) were detected. Albumin creatinine ratio (ACR) was calculated from albumin and creatinine measured in urine samples, and based on ACR, patients was classified as normoalbuminuria (ACR < 30 mg/g), microalbuminuria (ACR 30–299 mg/g), and macroalbuminuria (ACR ≥ 300 mg/g). Estimate glomerular filtration rate (eGFR) was calculated by the CKD-EPI creatinine equation [15] using the clinical data of patients.

### CGM parameters

Patients were asked to wear a continuous glucose monitoring system of Meiqi Company to monitor blood glucose levels every 5 min for 72 h continuously during the study. Capillary blood glucose at least four times was measured on each day of use of CGM to update the monitor. All 72 h of glucose data collected were calculated, TIR indicating the percentage of time that blood glucose was in the range of 3.9–10.0 mmol/L. Nocturnal TIR was defined as the percentage of time that blood glucose was in the range of 3.9–10.0 mmol/L between 00.00 am and 06:00 am (6 h). Patients were considered to be hypoglycaemic if they had at least one documented hypoglycaemic event during CGM monitoring. A blood glucose level < 3.9 mmol/L was recorded as one hypoglycaemic event. Nocturnal hypoglycaemic events are those that occur between 00.00 am and 06:00 am (6 h). Based on the original blood glucose data recorded by this system, a number of metrics concerning mean blood glucose (MBG) and glycemic variability (GV), including standard deviation (SD), average daily risk range (ADDR), mean amplitude of glucose excursions (MAGE), largest amplitude of plasma glucose excursions (LAGE), coefficient of variation (CV), and M-value were calculated using the EasyGV Version 9.0R2 provided by Oxford University.

### Statistical analysis

The SPSS 22.0 software package was used for statistical analysis. Continuous data for normal distributions were expressed as mean ± SD, while data for abnormal distributions were expressed as median (upper and lower quartiles). Categorical data were expressed as numbers (percentages). Two normally distributed samples were compared using the Student’s t-test. One-way ANOVA was used for multi-sample comparisons and the KruskalWallis test was used for abnormal distributions. Categorical variables were tested using the χ2 test. The Spearman analysis was applied to analyze the relationship between TIR (or nocturnal TIR) and ACR. The binary logistic regression was applied to examine the independent connection between TIR (or nocturnal TIR) and ACR by adjusting age, sex, diabetes duration, BMI, lipid situation, SBP, SCr, HbA1c (%), and GV metrics. In addition, the multinomial logistic regression was applied to examine the independent connection between TIR (or nocturnal TIR) and different stages of ACR by adjusting age, P < 0.05 was considered statistically significant.

## Results

### Clinical characteristics among stages of ACR Groups

The median (upper and lower quartiles) age of all 823 patients was 56.0[38.0,65.0] years, the median (upper and lower quartiles) duration of diabetes was 7[2, 12] years. There were 570 diabetic individuals without albuminuria (normoalbuminuria), 188 individuals with microalbuminuria and 65 diabetic individuals with macroalbuminuria. The prevalences of microalbuminuria and macroalbuminuria were 22.8% and 7.9%, respectively. With the aggravation of albuminuria, patients showed increased levels of SCr, SBP, MBG, ADDR, and M-value (P < 0.05), and lower levels of nocturnal TIR (P < 0.001). Median (upper and lower quartiles) nocturnal TIR was 97.50%(71.18%, 100.00%), 88.61%(56.88%, 100.00%), and 79.86%(47.85%, 99.23%) in normoalbuminuria (ACR < 30 mg/g), microalbuminuria (ACR 30–299 mg/g), and macroalbuminuria, respectively. TIR was significant different with the aggravation of albuminuria (P < 0.001). However, no significant difference between microalbuminuria and macroalbuminuria was evident(P = 0.98). In addition, there was no significant difference in HbA1c (%) among different groups.

### The comparison of clinical characteristics by quartiles (Q1-Q4) of TIR

Further analysis after dividing patients into groups was done according to quartiles of TIR((Q1):≤ 42.92%; (Q2): 42.92–69.13%; (Q3): 69.13–85.45%; (Q4): >85.45%). The characteristics were shown in Table 1. Patients with the highest quartiles of TIR had lower TC, TG, HbA1c (%), ACR, SD, MAGE, MBG, ADDR, CV and M-value (P < 0.001). Notably, there was no significant difference in eGFR between the different TIR groups (P = 0.702).

**Table 1:** The comparison of clinical characteristics by quartiles (Q1-Q4) of TIR.

	Quartiles (Q1-Q4) of TIR					
	Q1	Q2	Q3	Q4	χ2/t/z	P
N	206	206	206	205		
Age (y)	56.0(47.5,65.0)	57.0(48.0,66.0)	54.0(44.3,63.0)	56.0(48.0,64.0)	4.247	0.236
Diabetes duration (y)	8(3,15)	10(4,15)	6(2,12)	5(1,10)	31.323	< 0.001
Male (n, %)	66	63	70	72	3.834	0.28
SCr (µmol/L)	54.00(46.00,67.50)	56.00(46.00,67.00)	58.00(48.00,68.75)	59.00(48.00,70.00)	4.622	0.202
TC (mmol/L)	4.49(3.77,5.39)	4.46(3.72,5.35)	4.29(3.73,5.06)	4.27(3.53,4.88)	12.611	0.006
TG (mmol/L)	1.74(1.15,2.84)	1.59(1.59,2.56)	1.47(1.1,2.17)	1.37(1.02,2.12)	12.11	0.007
HDL (mmol/L)	0.97(0.86,1.19)	1.06(0.92,1.24)	1.06(0.9,1.24)	1.05(0.9,1.19)	8.794	0.032
LDL (mmol/L)	2.54(1.96,3.32)	2.66(2.07,3.37)	2.6(2.07,3.18)	2.51(1.89,3.12)	5.211	0.157
BMI	24.54(22.60,27.46)	25.34(22.0,27.24)	25.39(23.14,27.68)	25.39(22.60,27.78)	5.891	0.117
SBP (mmHg)	128(120,142)	128(120,140)	130(120,140)	129(120,138)	0.886	0.829
HbA1C (%)	9.9(8.9,11.7)	9(7.8,10.4)	7.9(7,9.2)	7(6.3,8.1)	256.012	< 0.001
eGFR, mL/min per 1.73 m2	107.74(98.50,121.56)	107.34(98.28,118.58)	111.59(100.4,123.42)	110.50(99.86,119.56)	1.417	0.702
ACR, mg/g	16.7(8.8,67.7)	13.7(7.5,59.0)	11.2(5.6,31.8)	10.4(5.7,31.3)	20.556	< 0.001
SD (mmol/L)	2.94(2.3,3.84)	2.79(2.29,3.38)	2.23(1.86,2.63)	1.49(1.27,1.8)	454.174	< 0.001
MAGE (mmol/L)	5.07(3.79,6.25)	5.12(4.06,6.21)	4.31(3.54,5.29)	3.38(2.89,4.04)	192.068	< 0.001
MBG (mmol/L)	11.96(11.25,13.04)	9.8(9.42,10.26)	8.46(8.17,8.86)	7.39(6.84,7.81)	793.17	< 0.001
CV	0.25(0.19,0.3)	0.28(0.24,0.35)	0.26(0.22,0.31)	0.2(0.17,0.25)	154.467	< 0.001
ADDR (mmol/L)	36.31(29.84,45.34)	36.31(29.84,45.34)	19.26(15.87,23.22)	11.71(8.75,14.46)	636.16	< 0.001
M-value (mmol/L)	24.72(16.64,34.93)	10.3(8.26,14.1)	4.71(3.77,5.95)	1.81(1.11,2.52)	784.969	< 0.001

TIR: time in range; Nocturnal TIR: nocturnal time in range; HbA1C: hemoglobin A1C; SD: standard deviation; MAGE: mean amplitude of glucose excursions; CV: coefficient of variation; ADDR: average daily risk range; eGFR: estimated glomerular filtration rate

### Prevalence of all stages of albuminuria in different quartiles (Q1-Q4) of TIR

**Fig. 1:**
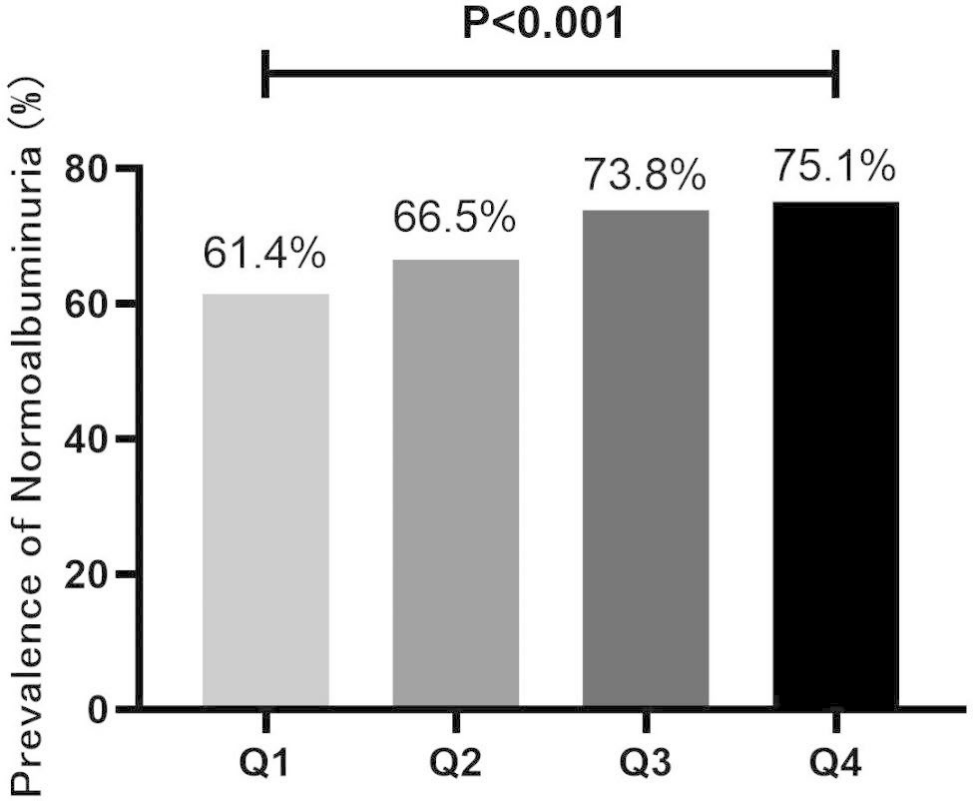
Prevalence of “Normalbuminuria” in different quartiles (Q1-Q4) of TIR. aTIR (Q1):≤ 42.92%; (Q2): 42.92–69.13%; (Q3): 69.13–85.45%; (Q4): >85.45%. bAs shown in this figure, patients were divided into groups according to quartiles of the time in range (TIR), the proportion of “Normoalbuminuria” increased with the increase of TIR (P < 0.001). P value for the significant Difference among the groups was determined by χ2-test

**Fig. 2:**
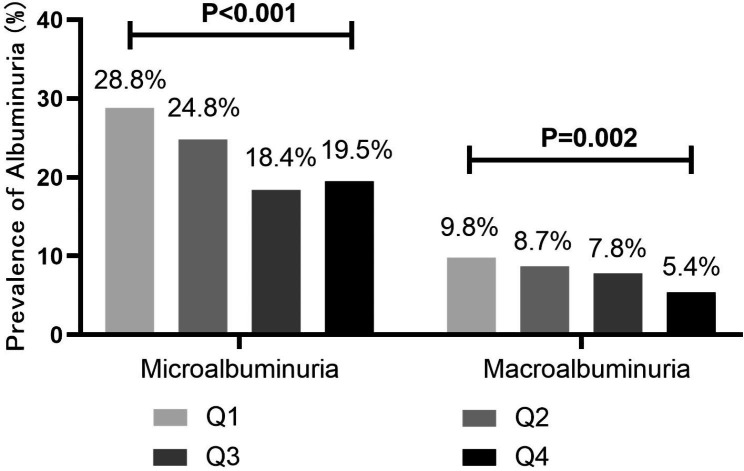
Prevalence of Albuminuria in different quartiles (Q1-Q4) of TIR. aTIR (Q1):≤ 42.92%; (Q2): 42.92–69.13%; (Q3): 69.13–85.45%; (Q4): >85.45%. bAs shown in this figure, patients were divided into groups according to quartiles of the time in range (TIR), the proportion of microalbuminuria and macroalbuminuria decreased with the increase of TIR (P < 0.05). P value for the significant difference among the groups was determined by χ2-test

As shown in Fig. 1, individuals were classified into groups according to quartiles of the TIR, the proportion of “Normoalbuminuria” increased with the increase of TIR (P < 0.001). What else, the proportion of microalbuminuria and macroalbuminuria decreased with the increase of TIR (P < 0.05) (Fig. 2).

### The correlation of TIR and nocturnal TIR with HbA1c, eGFR and ACR

Spearman analysis was used to analyse the correlation between TIR and nocturnal TIR with HbA1c, eGFR and ACR and the results are shown in Table 2. Both TIR and nocturnal TIR were negatively correlated with HbA1c (P < 0.001, P < 0.001), ACR (P < 0.001, P < 0.001) and insignificantly correlated with eGFR (P = 0.367,P = 0.495). Notably, the correlation coefficient between nocturnal TIR and ACR was higher than that of TIR (R=-0.159 vs. R=-0.147).

**Table 2:** The Correlation of TIR and nocturnal TIR with HbA1c, eGFR and ACR.

		HbA1c	eGFR	ACR
TIR (3.9–10 mmol/L)	R	-0.544	0.029	-0.147
	P	< 0.001	0.367	< 0.001
Nocturnal TIR (3.9–10 mmol/L)	R	-0.425	0.022	-0.159
	P	< 0.001	0.495	< 0.001

TIR: time in range; Nocturnal TIR: nocturnal time in range; HbA1C: hemoglobin A1C; ACR: albumin/creatinine ratio; eGFR: estimated glomerular filtration rate

### Associations between TIR, nocturnal TIR and ACR

Association of TIR and nocturnal TIR with the presence of albuminuria were investigated using binary logistic regression (Tables 3 and 4). After adjusting for age, diabetes duration, sex, BMI, lipid profile, blood pressure, and HbA1c (%) (model 1), the data revealed that TIR (odds ratio(OR): 0.992, 95% confidence interval (CI): 0.985–0.999, P = 0.026) as well as nocturnal TIR (OR: 0.990, 95% CI: 0.984–0.996, P = 0.002) was obviously related to the presence of albuminuria. After adjusting for SD, MAGE, CV and ADDR (model 2,3,4,5), the association persisted. However, after adjustment of M-value (model 6), the link between albuminuria and TIR was weakened (p = 0.197), the association between the presence of albuminuria and nocturnal TIR was still strong (p = 0.015).

**Table 3:** Associations between between TIR and albuminuria

	Microalbuminuria		Macroalbuminuria		Any Albuminuria	
	OR (95%CI)	P	OR (95%CI)	P	OR (95%CI)	P
Model 1						
TIR	0.993(0.985-1.000)	0.062	0.988(0.976-1.000)	0.053	0.992(0.985–0.999)	0.026
Model 2						
TIR	0.994(0.986–1.002)	0.126	0.99(0.977–1.002)	0.112	0.993(0.986–1.001)	0.082
SD	1.069(0.926–1.233)	0.363	1.113(0.941–1.316)	0.213	1.091(0.955–1.247)	0.2
Model 3						
TIR	0.989(0.981–0.997)	0.008	0.987(0.974-1)	0.052	0.989(0.981–0.996)	0.004
MAGE	0.91(0.821–1.008)	0.072	0.948(0.816–1.101)	0.484	0.92(0.838–1.01)	0.079
Model 4						
TIR	0.993(0.985-1)	0.065	0.987(0.975-1)	0.05	0.992(0.985–0.999)	0.026
CV	0.626(0.086–4.558)	0.644	13.67(1.45-128.892)	0.022	2.168(0.437–10.764)	0.344
Model 5						
TIR	0.983(0.973–0.994)	0.003	0.988(0.973–1.003)	0.118	0.986(0.977–0.996)	0.006
ADDR	0.972(0.95–0.994)	0.014	1(0.975–1.025)	0.989	0.983(0.965–1.002)	0.078
Model 6						
TIR	0.994(0.985–1.003)	0.216	0.992(0.979–1.005)	0.224	0.995(0.986–1.003)	0.197
M-value	1.005(0.992–1.017)	0.482	1.01(0.998–1.022)	0.105	1.007(0.997–1.018)	0.164

a TIR: time in range; HbA1C: hemoglobin A1C; SD: standard deviation; MAGE: mean amplitude of glucose excursions; CV: coefficient of variation; ADDR: average daily risk range; CI: confidence interval

b Model 1 was adjusted for age, diabetes duration, sex, BMI, lipid profile, blood pressure, and HbA1c (%); model 2 was adjusted for variables as in model 1 and for SD; model 3 was adjusted for variables as in model 1 and for MAGE; model 4 was adjusted for variables as in model 1 and for CV; model 5 was adjusted for variables as in model 1 and for ADDR; model 6 was adjusted for variables as in model 1 and for M-value

The multinomial Logistic regression found the weak relationship between TIR and severe stage of albuminuria (Microalbuminuria: P = 0.062. Macroalbuminuria: P = 0.053.) (Tables 4 and 6) (model 1). However, nocturnal TIR was obviously related to the severity of albuminuria (Microalbuminuria: OR: 0.992, 95% CI: 0.985–0.998, P = 0.015. Macroalbuminuria: OR: 0.984, 95% CI: 0.974–0.994, P = 0.002.) (model 1). Furthermore, the association persisted after adjusting for SD, MAGE, CV and ADDR (model 2,3,4,5), after adjustment of M-value (model 6), the link between the severe stage of albuminuria and nocturnal TIR was weakened (Microalbuminuria: p = 0.051; Macroalbuminuria: P = 0.012).

**Table 4:** Associations between between nocturnal TIR and albuminuria

	Microalbuminuria		Macroalbuminuria		Any Albuminuria	
	OR (95%CI)	P	OR (95%CI)	P	OR (95%CI)	P
Model 1						
Nocturnal TIR	0.992(0.985–0.998)	0.015	0.984(0.974–0.994)	0.002	0.990(0.984–0.996)	0.002
Model 2						
Nocturnal TIR	0.992(0.985–0.999)	0.027	0.992(0.985–0.999)	0.027	0.991(0.984–0.997)	0.002
SD	1.068(0.931–1.225)	0.348	1.068(0.931–1.225)	0.348	1.087(0.956–1.236)	0.002
Model 3						
Nocturnal TIR	0.99(0.983–0.997)	0.005	0.983(0.972–0.993)	0.002	0.988(0.982–0.995)	0.001
MAGE	0.928(0.842–1.024)	0.137	0.954(0.827–1.101)	0.522	0.935(0.856–1.022)	0.139
Model 4						
Nocturnal TIR	0.992(0.985–0.999)	0.018	0.984(0.973–0.994)	0.002	0.99(0.983–0.996)	0.001
CV	0.72(0.1-5.183)	0.744	13.837(1.455-131.599)	0.022	2.392(0.487–11.761)	0.283
Model 5						
Nocturnal TIR	0.988(0.98–0.996)	0.003	0.984(0.972–0.996)	0.007	0.987(0.98–0.995)	0.001
ADDR	0.981(0.963–0.999)	0.04	0.998(0.975–1.021)	0.867	0.987(0.972–1.003)	0.109
Model 6						
Nocturnal TIR	0.992(0.985-1)	0.051	0.986(0.975–0.997)	0.012	0.991(0.985–0.998)	0.015
M-value	1.003(0.991–1.014)	0.648	1.008(0.998–1.018)	0.128	1.005(0.997–1.014)	0.234

a TIR: time in range; Nocturnal TIR: nocturnal time in range; HbA1C: hemoglobin A1C; SD:standard deviation; MAGE: mean amplitude of glucose excursions; CV: coefficient of variation; ADDR: average daily risk range; CI: confidence interval; eGFR: estimated glomerular filtration rate

b Model 1 was adjusted for age, diabetes duration, sex, BMI, lipid profile, blood pressure, and HbA1c (%); model 2 was adjusted for variables as in model 1 and for SD; model 3 was adjusted for variables as in model 1 and for MAGE; model 4 was adjusted for variables as in model 1 and for CV; model 5 was adjusted for variables as in model 1 and for ADDR; model 6 was adjusted for variables as in model 1 and for M-value

**Table 5:** The Correlation of hypoglycaemic events and nocturnal hypoglycaemic events with HbA1c, eGFR and ACR.

		HbA1c	eGFR	ACR
Hypoglycaemic events	R	-0.138	-0.131	-0.023
	P	< 0.001	< 0.001	0.476
Nocturnal hypoglycaemic events	R	-0.091	-0.096	-0.030
	P	0.005	0.003	0.351

HbA1C: hemoglobin A1C; ACR: albumin/creatinine ratio; eGFR: estimated glomerular filtration rate

**Table 6:** The correlation between eGFR and the number of hypoglycaemic events

	B	95%CI	p
number of hypoglycaemic events	-0.090	(-2.769, -0.761)	0.001
age	-0.497	(-1.103, -0.876)	< 0.001
duration of diabetes	-0.149	(-0.774, -0.347)	< 0.001
blood pressure	-0.073	(-0.188, -0.033)	0.005

### The correlation of hypoglycaemic events and nocturnal hypoglycaemic events with HbA1c, eGFR and ACR

The number of nocturnal hypoglycaemic events was used as a statistic for hypoglycaemia. Based on Spearman analysis, Table 5 presents the correlations between hypoglycaemicevents and nocturnal hypoglycaemic events with HbA1c, eGFR, and ACR. Hypoglycaemia and nocturnal hypoglycaemia were both negatively correlated with HbA1c (P < 0.001, P = 0.005), eGFR (P < 0.001, P = 0.003), and not significantly correlated with ACR (P = 0.476, P = 0.351). A multiple linear regression model was used to assess the correlation between eGFR and the number of hypoglycaemic events. The model showed that the number of hypoglycaemic, age, duration of diabetes and blood pressure were independent risk factors for eGFR. (B = -0.090, -0.497, -0.149, -0.073, all P < 0.05, Table 6).

## Discussion

In our cohort of 823 T2D patients, the prevalence of albuminuria decreased with increasing TIR quartiles. Binary logistic regression revealed that TIR as well as nocturnal TIR was obviously related to the presence of albuminuria after adjusting for age, diabetes duration, sex, BMI, lipid profile, blood pressure, HbA1c (%), SD, MAGE, CV and ADDR. However, after adjustment of M-value, the link between albuminuria and TIR was weakened, the association between the presence of albuminuria nocturnal TIR was still significant. Multiple regression found the relationship between TIR and severity of albuminuria. However, nocturnal TIR was obviously related to the severe stage of albuminuria regardless of SD, MAGE, CV and ADDR. In our study, eGFR was not significantly associated with TIR, but was significantly associated with the number of hypoglycemic events and the number of nocturnal hypoglycemic events.

As the gold standard for assessing glycaemic management, hemoglobin A1c (HbA1c) is considered to have a strong correlation with the microvascular complications of diabetes, including diabetic nephropathy [16, 17]. However, HbA1c does not reflect information on hyperglycaemia, hypoglycaemia and fluctuations in blood glucose, nor does it reflect the magnitude and frequency of intra- and inter-day glucose changes [18, 19]. In addition, the measurement of glycated haemoglobin can be affected by specific conditions including kidney insufficiency, anaemia, pregnancy, haemoglobinopathies and iron deficiency [20–23]. Due to the shortcomings of HbA1c, CGM-derived indicators, especially TIR has become an alternative marker of glycemic control over the last few years [24, 25].

There is insufficient evidence in previous studies to demonstrate the association of glycaemic variability with diabetic nephropathy in T2DM. The study by S.-M. Jin et al. did not find an independent association between glycaemic variability and the degree of proteinuria in patients with type 2 diabetes [26]. Subramanian S et al. suggest that glycaemic variability may be a factor in the development of DKD, but clear evidence is lacking [27]. Wakasugi S et al. demonstrated that FLP-CGMderived metrics related to intraday and interday glucose variability, including TIR, SD, MAGE and MODD, were significantly associated with albuminuria severity, and these associations remained significant after adjustment for HbA1c [28]. Our study also found that TIR was obviously related to the presence of albuminuria after adjusting for glycaemic variability Indicators including SD, MAGE, CV and ADDR. However, in contrast to their results, multiple regression analysis found a weaker relationship between TIR and the stage of albuminuria severity.

A series of studies have demonstrated a correlation between CGM indicators, represented by TIR, and the development or progression of albuminuria in T2DM. Yoo JH et al. demonstrated that CGM-derived TIR significantly associated with the risk of albuminuria, even after adjusting for various confounding factors including CV. With a 10% increase in TIR, the risk of albuminuria was reduced by 6% [9]. However, after further adjustment for HbA1c, the study did not show a significant association between TIR and albuminuria. In contrast to their results, we found that TIR as well as nocturnal TIR was obviously related to the presence of albuminuria even after adjustment for HbA1c. In the study by Varghese JS, participants were divided into three groups based on glycemic profile (‘TIR profile’, ‘hyper’ and ‘hypo’). Compared with ‘TIR profile’, both ‘hyper’ and ‘hypo’ profiles had higher odds of macroalbuminuria and higher odds of diabetic kidney disease [29]. TIR was demonstrated to be associated with UACR, DPN and T2DM duration in the multicentre, prospective cohort study by Kuroda N et al. [30]. Consistent with those fndings, we found that the prevalence of albuminuria decreased as the TIR quartiles increased. The independently negative association between TIR and the presence of albuminuria exists even after adjusting for several risk factors including HbA1c.

There is little previous research on the relationship between nocturnal blood glucose and diabetic complications. A study of T1DM showed that reduced nocturnal TIR was more closely associated with function of sudomotor nerves function of sudomotor nerves [31]. In our study, nocturnal TIR showed a better correlation with albuminuria compared to TIR. Binary logistic regression analysis showed that TIR as well as nocturnal TIR were significantly correlated with the presence of albuminuria, after adjustment for a range of indicators. However, after further correction for M-value, only nocturnal TIR remained significant. Furthermore, multiple regression analysis showed that TIR did not correlate significantly with the severity of albuminuria, while nocturnal TIR correlated significantly, even after adjustment for a number of indicators including HbA1c. One possible explanation is attributed to the effect of growth hormone secreted at night on the kidneys. Growth hormone (GH) is widely used to treat short stature in children, including children with chronic kidney disease (CKD). GH-excess can affect kidney health by causing glomerular hyperfiltration, hypertrophy, and glomerulosclerosis [32]. GH-excess is also an important promoter of diabetes nephropathy in T1DM patients. Studies in patients with T1DM showed that urinary GH and IGF-1 levels were related to microalbuminuria in patients [33]. In addition, GH also has a renal protective effect on humans. Studies have shown that GH therapy can protect cisplatin-induced nephropathy in rats [34]. To sum up, GH secreted at night has an impact on blood glucose and kidneys, and the nocturnal TIR can better include its impact on blood glucose, which may explain the better correlation between nocturnal TIR and albuminuria. In addition, cortisol as a circadian hormone is not easy to ignore. a study by Roy et al. [35] found a tendency for elevated cortisol secretion in patients with diabetic retinopathy or diabetic cardiovascular complications. Chiodini I et al. [36] showed that the degree of midnight cortisol secretion was directly related to the presence and number of complications of type 2 diabetes, including DKD. In contrast, other parameters of cortisol secretion were not significantly correlated with the presence or number of diabetic complications. We can therefore speculate that nocturnal TIR could incorporate the effect of nocturnal cortisol levels on blood glucose and therefore correlate better with diabetic nephropathy. In addition, diabetes significantly affects melatonin secretion levels at night. Hikichi et al. [37] compared melatonin secretion at night and during the day in non-diabetic and diabetic subjects and found that melatonin levels were lower at night in patients with diabetes, but not during the day as affected by diabetes. The study by Baris Afsar et al. [38] suggests that melatonin activates cardiovascular system and renal receptors to protect DN in preclinical models. Considering that elevated blood glucose at night may inhibit melatonin secretion, thus depriving the kidneys of the protective effect of melatonin, the association between nocturnal TIR and proteinuria is more significant compared to full-day TIR.

Unlike albuminuria and TIR, our study found no significant correlation between eGFR and TIR. In recent years, the role of the renal tubules has received increasing attention in studies on the pathogenesis of DKD. As a traditional indicator of kidney disease, the increase of microalbuminuria or creatinine based glomerular filtration rate (eGFR) may occur long after the decline of renal function [39]. Some biomarkers of proximal renal tubular injury, such as kidney injury molecule 1 (KIM-1), N-acetyl-b-D-glucosaminidase (NAG), and liver fat acid binding protein (L-FABP), appear abnormal before proteinuria, and may become new biomarkers for DKD prediction [40]. At present, it is believed that the mechanism of albuminuria is the excessive inflammatory reaction of proximate tubular epithelial cells (PTECs) under the condition of diabetes [41, 42]. Considering the central role of renal tubules rather than glomeruli in the occurrence and development of early stage DKD, it can be explained why TIR, as a blood glucose control indicator, has a good correlation with albuminuria but not with eGFR. A notable result was that hypoglycaemic and nocturnal hypoglycaemic events were negatively correlated with eGFR. According to the study by Khanimov I [43], eGFR was strongly associated with an increased incidence of hypoglycaemia during hospitalization in non-critically ill patients. However, this study also showed that renal function was a strong predictor of hypoglycaemia regardless of the presence of diabetes, implying that hypoglycaemic events may not be specific for the prediction of diabetic nephropathy.

Our study had several limitations that should be noted. Firstly, this is a retrospective, single-centre study and it can only describe the correlation, not the causal relationship. Secondly, all subjects received CGM for 72 h, which may not be representative of overall glucose status. Some glucose-raising hormones that may play a role in the development of diabetic complications such as cortisol, growth hormone and melatonin were not measured.

In conclusion, our study revealed that TIR and nocturnal TIR is associated with the presence and severity of albuminuria independent of HbA1c and GV metrics. Nocturnal TIR shows better correlation than TIR. Hypoglycemia and nocturnal hypoglycemia events were negatively correlated with eGFR, while TIR was not correlated with eGFR. The role of TIR and nocturnal TIR in the evaluation of diabetes kidney disease should be emphasized.

## Data Availability

The datasets used and analyzed during the current study are available from the corresponding author on reasonable request.

## Abbreviations

TIR: time in range
T2DM: type 2 diabetes mellitus
eGFR: estimated glomerular filtration rate
GV: glycemic variability
CV: cardiovascular
HbA1C: hemoglobin A1C
CGM: continuous glucose monitoring
SBP: systolic blood pressure
DBP: diastolic blood pressure
BMI: body mass index
TC: total cholesterol
TG: triglyceride
HDL: high-density lipoprotein
LDL: low-density lipoprotein
Scr: serum creatinine
ACR: urinary albumin/creatinine ratio
DKD: diabetic kidney disease
DR: diabetic retinopathy
MBG: mean blood glucose.
SD: standard deviation.
MAGE: mean glucose fluctuation amplitude
CV: coefficient of variation
MODD: mean absolute difference of daytime glucose
ADDR: mean daily risk range
TBR: time below range
TAR: time above range
OR: odds ratio
CI: confidence interval
T1DM: type 1 diabetes mellitus
